# Sex-specific differences in the efficacy of traditional low frequency versus high frequency spinal cord stimulation for chronic pain

**DOI:** 10.1186/s42234-022-00090-2

**Published:** 2022-04-28

**Authors:** Rosalynn R. Z. Conic, Jacob Caylor, Christina L. Cui, Zabrina Reyes, Eric Nelson, Sopyda Yin, Imanuel Lerman

**Affiliations:** 1 Department of Family Medicine and Public Health, University of California, San Diego, La Jolla, CA USA; 2 Department of Anesthesiology, Center for Pain Medicine, University of California San Diego School of Medicine, La Jolla, CA USA; 3 Northwest Pain Care, PS, Spokane, WA USA; 4 Division of Vascular and Endovascular Surgery, Department of Surgery, Duke University Medical Center, Durham, NC USA; 5 School of Medicine, University of California, San Diego, San Diego, CA USA; 6 College of Osteopathic Medicine of the Pacific Western University of Health Sciences, Pomona, CA USA; 7 Department of Electrical and Computer Engineering, University of California San Diego, La Jolla, CA USA; 8 VA Center of Excellence for Stress and Mental Health, VA San Diego Healthcare System, La Jolla, CA USA; 9 Affiliate Electrical and Computer Engineering, VA San Diego Healthcare System, Center for Stress and Mental Health, Center for Pain Medicine, UC San Diego Health, Qualcomm Institute, California Institute for Telecommunications and Information Technology (Calit2), VA San Diego, 3350 La Jolla Village Dr, (MC116A), San Diego, CA 92161 USA

**Keywords:** Spinal cord stimulator, Outcomes, HF-SCS, LF-SCS, VAS, Opioid, Sex

## Abstract

**Introduction:**

Spinal cord stimulation (SCS), an FDA-approved therapy for chronic pain, uses paresthesia (low frequency SCS (LF-SCS)) or paresthesia-free (such as high-frequency SCS (HF-SCS)) systems, providing analgesia through partially-elucidated mechanisms, with recent studies indicating a sexual dimorphism in pain pathogenesis (Bretherton et al., Neuromodulation, 2021; Paller et al., Pain Med 10:289–299, 2009; Slyer et al., Neuromodulation, 2019; Van Buyten et al., Neuromodulation 20:642–649, 2017; Mekhail et al., Pain Pract, 2021). We aim to evaluate SCS therapy sex effects based on paradigm, utilizing visual analog scores (VAS), perceived pain reduction (PPR), and opioid use.

**Methods:**

A retrospective cohort study of SCS patients implanted between 2004 and 2020 (*n* = 237) was conducted. Descriptive statistics and linear mixed methods analyses were used.

**Results:**

HF-SCS (10 kHz) was implanted in 94 patients (40 females, 54 males), and LF-SCS in 143 (70 females, 73 males). At 3 months and 6 months, HF-SCS (*p* < 0.001) and LF-SCS (*p* < 0.005) had lower VAS scores compared to baseline (*p* < 0.005), with no differences across groups. PPR improved in both post-implantation (*p* < 0.006) and at 3 months (*p* < 0.004 respectively), compared to baseline persisting to 6 (*p* < 0.003) and 12 months (*p* < 0.01) for HF-SCS, with significantly better PPR for HF-SCS at 3 (*p* < 0.008) and 6 (*p* < 0.001) months compared to LF-SCS. There were no differences in opioid use from baseline for either modality; however LF-SCS patients used more opioids at every time point (*p* < 0.05) compared to HF-SCS.

VAS was improved for all modalities in both sexes at 3 months (*p* = 0.001), which persisted to 6 months (*p* < 0.05) for HF-SCS males and females, and LF-SCS females. Female HF-SCS had improved PPR at 3 (*p* = 0.016) and 6 (*p* = 0.022) months compared to baseline, and at 6 (*p* = 0.004) months compared to LF-SCS. Male HF-SCS and LF-SCS had improved PPR post-implantation (*p* < 0.05) and at 3 months (*p* < 0.05), with HF-SCS having greater benefit at 3 (*p* < 0.05) and 6 (*p* < 0.05) months. LF-SCS males but not females used less opioids at 6 months (*p* = 0.017) compared to baseline; however this effect did not persist.

On linear mixed model analyses, including age, sex and stimulator type, VAS decreased with age, at each timepoint, and had a trend towards increasing with female sex, while PPR increased at 3 and 6 months and lastly HF-SCS was associated with decreased opioid use.

**Discussion:**

PPR at 3 and 6 months improved to a greater extent in HF-SCS. HF-SCS females had improved PPR at 3 and 6 months, and only LF-SCS males used less opioids at 6 months, potentially indicating sex-based pathway. Future studies should further elucidate differences in sex-based pathways and identify optimal SCS opioid-sparing paradigms for chronic pain patients.

**Supplementary Information:**

The online version contains supplementary material available at 10.1186/s42234-022-00090-2.

## Introduction

Chronic pain, which is defined as pain persisting for more than 6 months, affects one in five Americans and can lead to reduced mobility and function as well as depression anxiety and other psychosocial changes (Dahlhamer et al. 2018; Turk et al. 2010). The standard treatment is medical management with a combination of non-steroid anti-inflammatory drugs, muscle relaxants and opioids; however, given the high risk for adverse outcomes with long-term opioid therapy, other alternatives are needed (Chou et al. 2009; Volkow and McLellan 2016).

One promising potential therapeutic for chronic pain; spinal cord stimulation (SCS), is indicated for chronic pain in conditions including Failed Back Surgery Syndrome (FBSS), Complex Regional Pain Syndrome (CRPS) Type I and II, painful diabetic neuropathy, and intractable low back and leg pain, and has been used for postherpetic neuralgia, pain due to peripheral nerve injury, intercostal neuralgia and phantom limb pain (Dones and Levi 2018). SCS systems are implanted in the epidural space and deliver electrical pulses to decrease or block transmission of pain signals at spinal segmental and supraspinal levels (Kapural et al. 2015). In traditional SCS, also known as low-frequency SCS (LF-SCS) or paresthesia-based SCS, the electrical pulses range between 2 and 60 Hz, most often in the 40-60 Hz range (Kapural et al. 2015; Melzack and Wall 1965; Caylor et al. 2019). But LF-SCS can be limited by anatomical restrictions that reduce or abolish coverage to certain targets, such as lower back and foot, while patient intolerance of paresthesia and loss of efficacy over time is common (Kumar et al. 2006a). More recently, −the FDA approved high-frequency SCS (HF-SCS), a type of paresthesia-free SCS (PF-SCS), that delivers 10,000kHz pulses; it consistently improves pain relief (for up to 36 months) improves pain relief (for up to 36 months) (Al-Kaisy et al. 2018) and to a greater extent when compared to LF-SCS (for up to 2 years) (Kapural et al. 2015; Kapural et al. 2016). Both LF-SCS and HF-SCS implantation is associated with decreased opioid use (Kapural et al. 2015; Rapcan et al. 2015), albeit this may not be clinically significant among opioid experienced patients (Vu et al. 2022), reduced disability (Russo et al. 2016; North et al. 2016; Al-Kaisy et al. 2014), while others have shown and improved sleep (Al-Kaisy et al. 2014; Van Buyten et al. 2013). However, up to 40% of patients experience complications including: lead migration, lead fracture, pain at the site of the implanted genera- tor, infection, and more rarely dural puncture headache, cerebrospinal fluid leakage and epidural hemorrhage, that may require additional procedures (Mekhail et al. 2011; Eldabe et al. 2016).

**Fig. 1:**
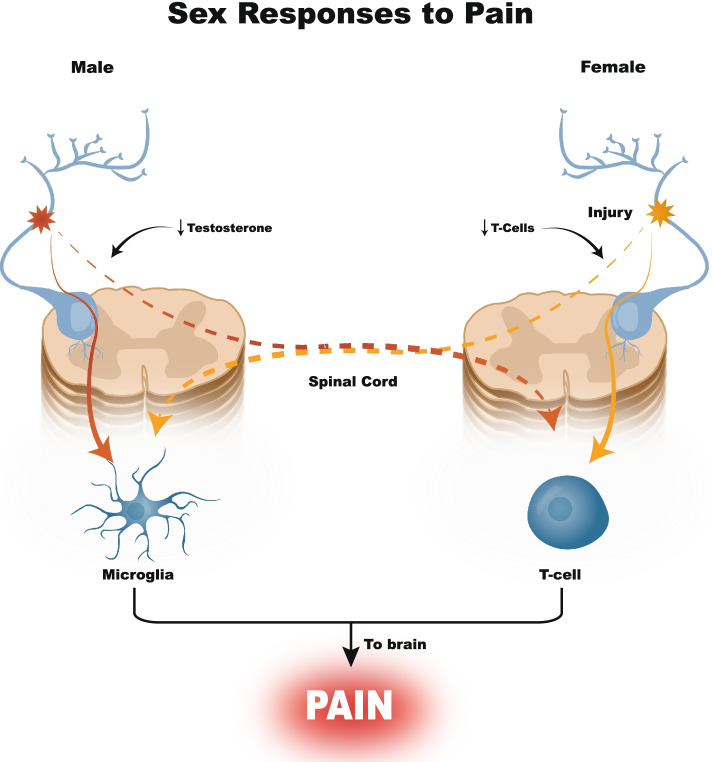
Sex-based differences in pain pathways. In the male model, microglia are activated to transmit pain, mediated by testosterone levels, while in the female model, the pain pathway is T-cell mediated. If a male has low testosterone levels, the pain pathway switches to the T-cell mediated pathway. Conversely, if a female takes exogenous testosterone, or if she has low T-cell levels, the pain pathway will switch to the microglia pathway

Given the recent discovery of sex-specific endogenous pain pathways (Fillingim et al. 2009), there is considerable interest in sex-based SCS efficacy. Preclinical models show that male allodynia can develop via a testosterone-dependent glial cell pathway (Sorge et al. 2011) while females utilize a T-cell-dependent mechanism (Fig. 1) (Sorge et al. 2015). Clinically, females respond differently to analgesics while they are more likely to have an SCS explanted due to inadequate pain relief compared to males; however, sex specific SCS response remains understudied (Bretherton et al. 2021; Paller et al. 2009; Slyer et al. 2019; Van Buyten et al. 2017). With few reports that identify interactions between sex and SCS we aimed to fill this knowledge gap through a single site (University of California San Diego), large (*n* = 237) retrospective (2004–2020) analyses that compared SCS paradigm (LF-SCS vs HF-SCS), efficacy (pain relief and opiate sparing effects) across sex (Mekhail et al. 2021).

## Methods

This study was approved by the institutional review board at the University of California San Diego (IRB#20031). A retrospective chart review of patients who underwent LF-SCS and HF-SCS trials and implantation at the University of California San Diego between October 2004 and February 2020 was performed (*n* = 237). Patients who did not undergo permanent implantation, patients without demographic data and those with multiple pain conditions requiring separate treatment were excluded. Data regarding patient age, sex, primary diagnosis, type of SCS implant, revision, and explant were collected. Outcome data were collected 3 months prior to trial (baseline), within 1–2 weeks after implantation (post-implantation) and at 3, 6, and 12 months consisted of visual analogue scores (VAS) for pain (rated from 0 to 10), perceived pain reduction attributable to the SCS (PPR, rated from 0 to 100 as a percentage), and opioid dosage converted into morphine equivalents (continuous) (Calculating Total Daily Dose of Opioids for Safer Dosage n.d.).

Chi square test and Fisher exact test were used to analyze categorical data. Paired t-test was used to compare the raw pain change between each time point and the baseline measurement. T-test was used to evaluate raw pain change between stimulator types across each time point. While the confidence intervals overlap for some variables which are statistically significant, the values represented in the tables and graphics are raw values rather than mean differences, and may overlap despite a statistically significant result (Greenland et al. 2016). Linear mixed models were used to account for repeated measures for each outcome. Results were stratified by type of stimulation paradigm (LF-SCS vs HF-SCS) and sex. R statistical software version 4.1.1 was used for data analysis (R Core Team 2020). The dataset analyzed in this study is available from the corresponding author on reasonable request.

## Results

There were 237 patients total, of whom 143 patients were implanted with an LF-SCS and 94 who were implanted with an HF-SCS (Table 1). Patients implanted with HF-SCS tended to be older (61.02 ± 14.48 vs 54.07 ± 15.05, *p* < 0.001, using t-test) and Non-Hispanic (84% vs 77.6%, *p* = 0.03). The most common primary indication for implant was FBSS in both groups (44.8% LF-SCS vs. 39.4% HF-SCS), followed by lumbar radiculopathy (21% LF-SCS vs. 21.3% HF-SCS), CRPS (16.1% LF-SCS vs. 7.4% HF-SCS) and non-surgical refractory back pain (1.4% LF-SCS vs 14.9% HF-SCS).

**Table 1:** Patient characteristics by type of stimulator

	HF-SCS	LF-SCS	*p*-value
n	94	143	
Sex = Male (%)	54 (57.4)	70 (49.0)	0.251
Age (mean (SD))	61.02 (14.48)	54.07 (15.05)	**< 0.001**
Ethnicity (%)			**0.027**
African American	0 (0.0)	2 (1.4)	
Caucasian	3 (3.2)	17 (11.9)	
Hispanic	9 (9.6)	5 (3.5)	
Non-Hispanic	79 (84.0)	111 (77.6)	
Unknown (Patient cannot or refuses to declare ethnicity)	3 (3.2)	8 (5.6)	
Marriage Status (%)			0.838
Married	60 (63.8)	89 (62.2)	
Other/Unknown	1 (1.1)	2 (1.4)	
Separated/Divorced	13 (13.8)	19 (13.3)	
Single	16 (17.0)	30 (21.0)	
Widowed	4 (4.3)	3 (2.1)	
Payor Name (%)			**< 0.001**
Medicare	30 (31.9)	23 (16.1)	
None/Unknown	49 (52.1)	84 (58.7)	
Other Government	8 (8.5)	3 (2.1)	
Private Insurance	7 (7.4)	28 (19.6)	
Workman’s Compensation	0 (0.0)	5 (3.5)	
Primary Diagnosis for Implant (%)			**< 0.001**
Complex Regional Pain Syndrome	7 (7.4)	23 (16.1)	
Cranial Neuropathy	2 (2.1)	5 (3.5)	
Failed Back Surgery Syndrome	37 (39.4)	64 (44.8)	
Lumbar Radiculopathy	20 (21.3)	30 (21.0)	
Non-Surgical Refractory Back Pain	14 (14.9)	2 (1.4)	
Neuropathic Pain	7 (7.4)	17 (11.9)	
Other Chronic Pain	7 (7.4)	2 (1.4)	
Revision or Explant (%)	25 (17.5)	15 (16.0)	0.897
Patient status (%)			**0.016**
Alive	78 (83.0)	120 (83.9)	
Deceased	0 (0.0)	9 (6.3)	
Unknown	16 (17.0)	14 (9.8)	

**Fig. 2:**
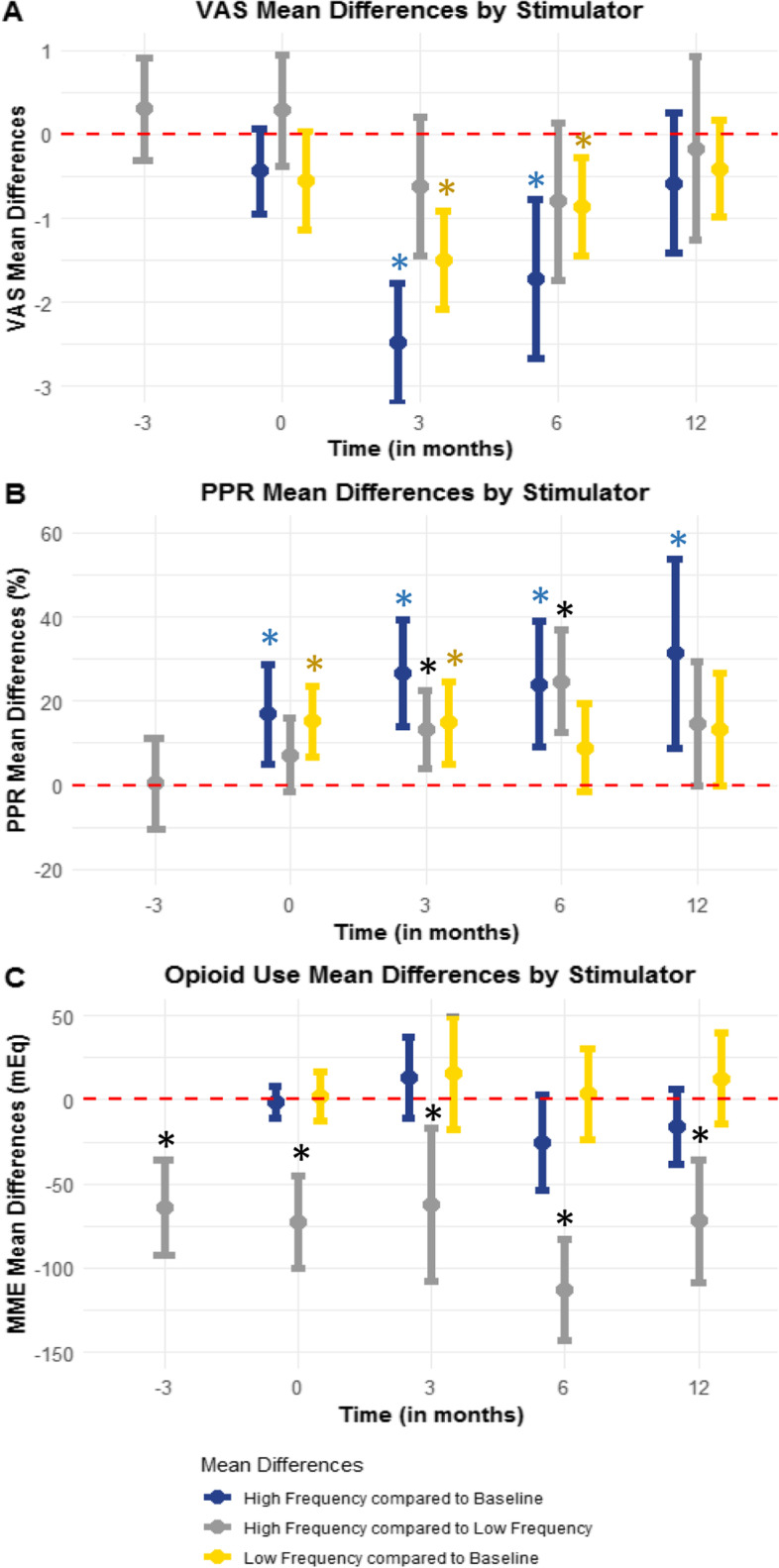
Mean Differences of Visual Analog Scale Pain Scores, Perceived Pain Reduction and Opioid Use based on Type of Spinal Cord Stimulator. Blue stars note significant difference for HF-SCS at that time point compared to baseline. Yellow stars note significant differences for LF-SCS at that time point compared to baseline. Black stars note significant differences between HF-SCS and LF-SCS at that time point. Confidence intervals which cross the dotted red line are considered nonsignificant. **a** Mean differences and 95% confidence intervals in visual analog scale pain scores by type of stimulator at baseline, post-implantation, 3 months after implant, 6 months after implant and 12 months after implant in comparison to baseline. **b** Mean differences and 95% confidence intervals in perceived pain reduction by type of stimulator at baseline, post-implantation, 3 months after implant, 6 months after implant and 12 months after implant in comparison to baseline. **c** Mean differences and 95% confidence intervals in opioid use using morphine miliequivalents by type of stimulator at baseline, post-implantation, 3 months after implant, 6 months after implant and 12 months after implant in comparison to baseline

HF-SCS and LF-SCS patients had significantly lower VAS compared to baseline at 3 (HF-SCS -2.48, 95%CI -3.19-(− 1.77), *p* < 0.001; LF-SCS -1.49, 95%CI -2.08-(− 0.91), *p* < 0.001) and 6 months (HF-SCS -1.72, 95%CI -2.67-(− 0.78), *p* < 0.001; LF-SCS -0.86, 95%CI -1.45-(− 0.27), *p* = 0.005) after implantation; however this effect did not survive to 12 months (HF-SCS *p* = 0.16, LF-SCS *p* = 0.15) and there were no differences across the two groups (3 months *p* = 0.18, 6 months *p* = 0.13, Fig. 2a, Tables 2 and 3, Supplemental Table 1, Supplemental Fig. 1).

**Table 2:** Comparison of mean differences and 95% confidence intervals of visual analog scale scores, perceived pain reduction and opioid use by between baseline and each individual time point based on stimulator type using paired t-test. For mean differences, the confidence interval is not considered significant if it crosses zero

	HF-SCS	***p***-value	LF-SCS	***p***-value
**Visual Analog Scale Pain Score (mean difference with 95% confidence interval)**				
Baseline-Post Implant	0.44(−0.95–0.07)	0.09	− 0.55(− 1.13–0.03)	0.061
Baseline- 3 Months Post Implant	−2.48(− 3.19-(− 1.77))	**< 0.001**	− 1.49(− 2.08-(− 0.91))	**< 0.001**
Baseline- 6 Months Post Implant	−1.72(− 2.67-(− 0.78))	**< 0.001**	− 0.86(− 1.45-(− 0.27))	**0.005**
Baseline- 12 Months Post Implant	−0.58(− 1.41–0.25)	0.16	−0.42(− 0.99–0.16)	0.15
**Perceived Pain Reduction (mean difference with 95% confidence interval)**				
Baseline-Post Implant	6.97(5.08–28.86)	**0.006**	15.18(6.61–23.70)	**< 0.001**
Baseline- 3 Months Post Implant	26.75 (14.02–39.48)	**< 0.001**	14.86(5.12–24.59)	**0.004**
Baseline- 6 Months Post Implant	24.0(9.0–39.0)	**0.003**	8.92(− 1.51–19.35)	0.09
Baseline- 12 Months Post Implant	31.36(8.88–53.85)	**0.01**	13.25(−0.15–26.65)	0.052
**Morphine Miliequivalents (mean difference with 95% confidence interval)**				
Baseline-Post Implant	−1.17(− 10.43–8.09)	0.99	1.84(− 12.77, 16.44)	0.8
Baseline- 3 Months Post Implant	13.4(− 10.69–37.48)	0.26	15.83(− 17.80, 49.46)	0.35
Baseline- 6 Months Post Implant	−25.18(− 53.43–3.06)	0.08	3.54(− 23.65, 30.74)	0.79
Baseline- 12 Months Post Implant	− 15.82(− 37.91–6.26)	0.15	12.73(− 14.62–40.08)	0.36

**Table 3:** Comparison of high frequency and low frequency spinal cord stimulator mean differences and 95% confidence intervals at each time point for visual analog scale pain scores, perceived pain reduction and opioid use. For mean differences, the confidence interval is considered not significant if it crosses zero

	HF-SCS compared to LF-SCS	***p***-value
**Visual Analog Scale Pain Score (mean difference with 95% confidence interval)**		
Baseline	0.30(−0.31–0.91)	0.26
Post Implant	0.28(−0.38–0.94)	0.47
3 Months Post Implant	−0.62(−1.44–0.20)	0.18
6 Months Post Implant	−0.8(− 1.73–0.13)	0.127
12 Months Post Implant	− 0.17(− 1.26–0.92)	0.81
**Perceived Pain Reduction (mean difference with 95% confidence interval)**		
Baseline	0.53(− 10.3–11.36)	0.99
Post Implant	7.24(−1.59–16.07)	0.09
3 Months Post Implant	13.25(4.04–22.46)	**0.008**
6 Months Post Implant	24.68(12.45–36.91)	**< 0.001**
12 Months Post Implant	14.67(−0.03–29.37)	0.067
**Morphine Miliequivalents (mean difference with 95% confidence interval)**		
Baseline	−63.99(−92.59-(−35.39))	**< 0.001**
Post Implant	−72.76(−100.41-(−45.11))	**< 0.001**
3 Months Post Implant	−62.6(− 108.08-(− 17.12))	**0.027**
6 Months Post Implant	−112.98(− 142.85-(− 83.11))	**< 0.001**
12 Months Post Implant	− 72.11(− 108.83-(− 35.39))	**0.014**

Both HF-SCS and LF-SCS had better PPR post-implantation (HF-SCS 6.97, 95%CI 5.08–28.86, *p* = 0.006; LF-SCS 15.18, 95%CI 6.61–23.70, *p* < 0.001) and at 3 months (HF-SCS 26.75, 95%CI 14.02–39.48, *p* < 0.001; LF-SCS 14.86 95%CI 5.12–24.59, *p* = 0.004) compared to baseline; however this effect was sustained to 6 (HF-SCS 24.0 95%CI 9.0–39.0, *p* = 0.003; LF-SCS 8.92, 95%CI -1.51-19.35, *p* = 0.09) and 12 months (HF-SCS 31.36, 95%CI 8.88–53.85, *p* = 0.01; LF-SCS 13.25, 95%CI -0.15-26.65, *p* = 0.052) only for the HF-SCS group. In addition, HF-SCS patients had significantly improved PPR compared to LF-SCS patients at 3 and 6 months (13.25, 95%CI 4.04–22.46 *p* = 0.008, 24.68, 95%CI 12.45–36.91 *p* < 0.001 respectively, Fig. 2b).

There were no differences in opioid use in HF-SCS or LF-SCS patients compared to baseline; however HF-SCS patients required significantly less opioids compared to LF-SCS at every time point (baseline − 63.99, 95%CI -92.59-(− 35.39), *p* < 0.001; post-implantation − 72.76, 95%CI -100.41-(− 45.11), *p* < 0.001; 3 months − 62.6, 95%CI -108.08-(− 17.12), *p* = 0.027; 6 months − 112.98, 95%CI -142.85-(− 83.11), *p* < 0.001 and 12 months − 72.11, 95%CI -108.83-(− 35.39), *p* = 0.014). Nine patients died during this study, all of whom were in the LF-SCS group. There were no differences in sex (49% LF-SCS vs. 57.4% HF-SCS, *p* = 0.25), marriage status (*p* = 0.84), and revisions or explants (16% LF-SCS vs. 17.5% HF-SCS, *p* = 0.89) between the two groups.

### Sex subset analysis

**Fig. 3:**
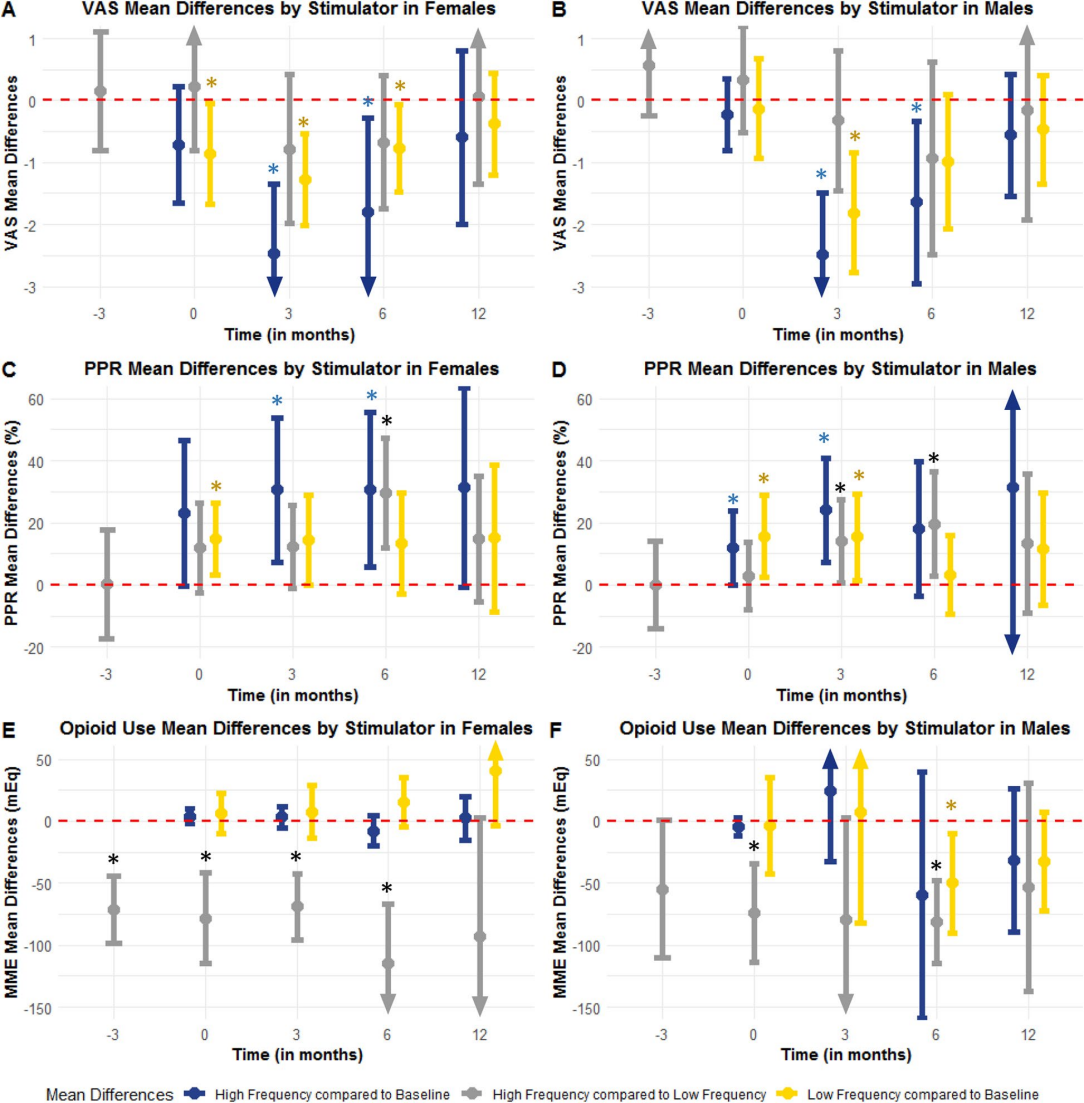
Visual Analog Scale Pain Scores, Perceived Pain Reduction and Morphine Equivalent Daily Dose based on Type of Spinal Cord Stimulator and Sex. Blue stars note significant difference for HF-SCS at that time point compared to baseline. Yellow stars note significant differences for LF-SCS at that time point compared to baseline. Black stars note significant differences between HF-SCS and LF-SCS at that time point. Confidence intervals which cross the dotted red line are considered nonsignificant. Arrows denote continuation of confidence interval out of graph limits. **a** Visual analog scale pain score distribution by type of stimulator at baseline, post-implantation, 3 months after implant, 6 months after implant and 12 months after implant in females. **b** Visual analog scale pain score distribution by type of stimulator at baseline, post-implantation, 3 months after implant, 6 months after implant and 12 months after implant in males. **c** Perceived pain reduction distribution by type of stimulator at baseline, post-implantation, 3 months after implant, 6 months after implant and 12 months after implant in females. **d** Perceived pain reduction distribution by type of stimulator at baseline, post-implantation, 3 months after implant, 6 months after implant and 12 months after implant in males. **e** Opioid use in morphine miliequivalents by type of stimulator at baseline, post-implantation, 3 months after implant, 6 months after implant and 12 months after implant in females. **f** Opioid use in morphine miliequivalents by type of stimulator at baseline, post-implantation, 3 months after implant, 6 months after implant and 12 months after implant in males

There were 73 females implanted with LF-SCS and 40 with HF-SCS (Table 4). The females implanted with HF-SCS tended to be older (53.70 ± 14.98 LF-SCS vs 65.03 ± 12.60 HF-SCS, *p* < 0.001). LF-SCS females had decreased VAS scores post-implantation (− 0.86, 95%CI -1.68-(− 0.04), *p* = 0.04) compared to baseline, and both HF-SCS and LF-SCS patients had decreased VAS at 3 (HF-SCS -2.47, 95%CI -3.6-(− 1.35), *p* < 0.001; LF-SCS -1.27, 95%CI -2.01-(− 0.53), *p* = 0.001) and 6 (HF-SCS -1.8, 95%CI -3.31-(− 0.29), *p* = 0.023; LF-SCS -0.77, 95%CI -1.47-(− 0.072), *p* = 0.032) months, which did not survive to 12 months (HF-SCS *p* = 0.37, LF-SCS *p* = 0.35), and there were no differences in VAS between HF-SCS and LF-SCS females (Fig. 3a, Tables 5 and 6, Supplemental Table 2, Fig. 2).

**Table 4:** Patient characteristics by type of stimulator and sex

	Female			Male		
	HF-SCS	LF-SCS	*p*-val	HF-SCS	LF-SCS	*p*-val
n	40	73		54	70	
Age (mean (SD))	65.03 (12.60)	53.70 (14.98)	**< 0.001**	58.06 (15.17)	54.46 (15.21)	0.193
Ethnicity (%)			0.62			**0.031**
African American	0 (0.0)	1 (1.4)		0 (0.0)	1 (1.4)	
Caucasian	2 (5.0)	5 (6.8)		1 (1.9)	12 (17.1)	
Hispanic	1 (2.5)	1 (1.4)		8 (14.8)	4 (5.7)	
Non-Hispanic	37 (92.5)	63 (86.3)		42 (77.8)	48 (68.6)	
Unknown (Patient cannot or refuses to declare ethnicity)	0 (0.0)	3 (4.1)		3 (5.6)	5 (7.1)	
Marriage Status (%)			0.73			0.79
Married	24 (60.0)	42 (57.5)		36 (66.7)	47 (67.1)	
Other/Unknown	0 (0.0)	0 (0.0)		1 (1.9)	2 (2.9)	
Separated/Divorced	8 (20.0)	14 (19.2)		5 (9.3)	5 (7.1)	
Single	5 (12.5)	14 (19.2)		11 (20.4)	16 (22.9)	
Widowed	3 (7.5)	3 (4.1)		1 (1.9)	0 (0.0)	
Insurance Type (%)			0.07			**0.018**
Medicare	15 (37.5)	12 (16.4)		15 (27.8)	11 (15.7)	
None/Unknown	19 (47.5)	39 (53.4)		30 (55.6)	45 (64.3)	
Other Government	1 (2.5)	1 (1.4)		7 (13.0)	2 (2.9)	
Private Insurance	5 (12.5)	18 (24.7)		2 (3.7)	10 (14.3)	
Workman’s Compensation	0 (0.0)	3 (4.1)		0 (0.0)	2 (2.9)	
Primary Diagnosis for Implant (%)			0.25			**0.006**
Complex Regional Pain Syndrome	4 (10.0)	13 (17.8)		3 (5.6)	10 (14.3)	
Cranial Neuropathy	1 (2.5)	2 (2.7)		1 (1.9)	3 (4.3)	
Failed Back Surgery Syndrome	18 (45.0)	32 (43.8)		19 (35.2)	32 (45.7)	
Lumbar Radiculopathy	8 (20.0)	15 (20.5)		12 (22.2)	15 (21.4)	
Non-Surgical Refractory Back Pain	5 (12.5)	1 (1.4)		9 (16.7)	1 (1.4)	
Neuropathic Pain	3 (7.5)	9 (12.3)		4 (7.4)	8 (11.4)	
Other Chronic Pain	1 (2.5)	1 (1.4)		6 (11.1)	1 (1.4)	
Revision or Explant (%)	6 (15.0)	17 (23.3)	0.42	9 (16.7)	8 (11.4)	0.564
Patient Status (%)			**0.036**			0.198
Alive	33 (82.5)	64 (87.7)		45 (83.3)	56 (80.0)	
Deceased	0 (0.0)	5 (6.8)		0 (0.0)	4 (5.7)	
Unknown	7 (17.5)	4 (5.5)		9 (16.7)	10 (14.3)

**Table 5:** Comparison of mean differences and 95% confidence intervals of visual analog scale scores, perceived pain reduction and opioid use by between baseline and each individual time point based on stimulator type and sex using paired t-test. For mean differences, the confidence interval is not considered significant if it crosses zero

	Female				Male			
	HF-SCS	***p***-value	LF-SCS	***p***-value	HF-SCS	***p***-value	LF-SCS	***p***-value
**Visual Analog Scale Pain Score (mean difference with 95% confidence interval)**								
Baseline-Post Implant	−0.72(− 1.66–0.22)	0.13	− 0.86(− 1.68-(− 0.04))	**0.04**	−0.23(− 0.81–0.35)	0.139	−0.13(− 0.94–0.67)	0.74
Baseline- 3 Months Post Implant	− 2.47(− 3.6-(− 1.35))	**< 0.001**	− 1.27(− 2.01-(− 0.53))	**0.001**	− 2.48(− 3.46-(− 1.5))	**< 0.001**	− 1.81(− 2.78-(− 0.84))	**< 0.001**
Baseline- 6 Months Post Implant	− 1.8(− 3.31-(− 0.29))	**0.023**	− 0.77(− 1.47-(− 0.072))	**0.032**	− 1.64(− 2.96-(− 0.33))	**0.018**	−0.98(− 2.07–0.1)	0.074
Baseline- 12 Months Post Implant	− 0.59(− 1.99–0.81)	0.37	−0.38(− 1.2–0.44)	0.35	−0.56(− 1.55–0.42)	0.22	−0.47(− 1.34–0.4)	0.27
**Perceived Pain Reduction (mean difference with 95% confidence interval)**								
Baseline-Post Implant	23.06(−0.3–46.43)	0.053	14.82(3.21–26.42)	**0.014**	11.84(0.03–23.65)	**0.049**	15.54(2.34–28.731)	**0.023**
Baseline- 3 Months Post Implant	30.5(7.17–53.83)	**0.016**	14.4(−0.2–29)	0.053	24.07(7.21–40.93)	**0.009**	15.33(1.38–29.29)	**0.033**
Baseline- 6 Months Post Implant	30.67(5.81–55.52)	**0.022**	13.33(−3.06–29.73)	0.11	18(− 3.74–39.74)	0.09	3.13(− 9.44–15.69)	0.6
Baseline- 12 Months Post Implant	31.43(−0.58–63.43)	0.053	15(−8.61–38.61)	0.18	31.25(−26.99–89.49)	0.19	11.5(−6.51–29.51)	0.18
**Morphine Miliequivalents (mean difference with 95% confidence interval)**								
Baseline-Post Implant	3.84(−2.21–9.89)	0.78	6.28(− 10.17–22.73)	0.45	−4.64(− 11.72–2.79)	0.25	−3.43(− 42.28–35.42)	0.86
Baseline- 3 Months Post Implant	3.21(− 5.11–11.53)	0.11	7.54(− 13.89–28.97)	0.48	24.62(− 32.36–81.59)	0.37	7.29(− 82.62–97.2)	0.87
Baseline- 6 Months Post Implant	− 7.9(− 20.13–4.33)	0.45	15.37(− 4.41–35.15)	0.12	−59.29(− 158.7–40.12)	0.21	−50.1(− 90.38-(− 9.83))	**0.017**
Baseline- 12 Months Post Implant	2.25(− 15.15–19.65)	0.83	40.35(− 3.73–84.42)	0.071	−31.79(− 89.43–25.86)	0.23	−32.38(− 71.96–7.2)	0.1

**Table 6:** Comparison of high frequency and low frequency spinal cord stimulator mean differences and 95% confidence intervals at each time point for visual analog scale pain scores, perceived pain reduction and opioid use according to sex. For mean differences, the confidence interval is considered not significant if it crosses zero

	Females		Males	
	HF-SCS compared to LF-SCS	***p***-value		***p***-value
**Visual Analog Scale Pain Score (mean difference with 95% confidence interval)**				
Baseline	0.15(− 0.8–1.1)	0.67	0.56(− 0.25–1.37)	0.13
Post Implant	0.22(− 0.81–1.25)	0.68	0.34(− 0.52–1.19)	0.52
3 Months Post Implant	−0.78(−1.98–0.42)	0.26	−0.32(− 1.45–0.81)	0.66
6 Months Post Implant	− 0.68(− 1.75–0.4)	0.32	−0.94(− 2.49–0.62)	0.25
12 Months Post Implant	0.07(− 1.34–1.47)	0.74	− 0.15(− 1.93–1.63)	0.99
**Perceived Pain Reduction (mean difference with 95% confidence interval)**				
Baseline	0.18(− 17.24–17.59)	0.96	− 0.09(− 14.07–13.89)	0.96
Post Implant	11.92(−2.64–26.48)	0.12	3.02(−7.82–13.87)	0.45
3 Months Post Implant	12.26(− 1.1–25.62)	0.12	14.18(0.77–27.58)	**0.034**
6 Months Post Implant	29.65(11.9–47.4)	**0.004**	19.6(2.88–36.32)	**0.027**
12 Months Post Implant	14.92(− 5.34–35.18)	0.22	13.33(− 9.13–35.79)	0.23
**Morphine Miliequivalents (mean difference with 95% confidence interval)**				
Baseline	−71.56(−98.48-(− 44.64))	**0.003**	− 55.09(− 110.62–0.44)	0.053
Post Implant	−78.33(− 114.69-(− 41.96))	**0.001**	− 73.93(− 113.57--34.29)	**0.006**
3 Months Post Implant	− 68.76(− 95.46-(− 42.07))	**0.006**	− 79.46(− 161.82–2.89)	0.19
6 Months Post Implant	−114.94(− 163.2-(− 66.68))	**0.01**	− 81.34(− 114.95--47.74)	**0.01**
12 Months Post Implant	−92.94(− 188.39–2.51)	0.064	−53.2(− 136.99–30.59)	0.21

Females with HF-SCS had greater PPR compared to baseline at 3 (HF-SCS 30.5, 95%CI 7.17–53.83, *p* = 0.016), and 6 months (HF-SCS 30.67, 95%CI 5.81–55.52, *p* = 0.022), without improvement at 12 months (*p* = 0.053, Fig. 3c). In addition, HF-SCS females had significantly better PPR scores at 6 months compared to LF-SCS females (29.65, 95%CI 11.9–47.4, *p* = 0.004).

While there were no differences in HF-SCS and LF-SCS females comparing each time point to baseline, HF-SCS females required significantly less opioids compared to LF-SCS females at baseline (− 71.56, 95%CI -98.48-(− 44.64), *p* = 0.003), post-implantation (HF-SCS -78.33, 95%CI -114.69-(− 41.96), *p* = 0.001), 3 months (− 68.76, 95%CI -95.46-(− 42.07), *p* = 0.006), and at 6 months (− 114.94, 95%CI -163.2-(− 66.68), *p* = 0.011), with a trend for lower opioid use for female HF-SCS patients at 12 months (− 92.94, 95%CI -188.39-2.51, *p* = 0.064; Fig. 3e). There were no differences in ethnicity (*p* = 0.62), marriage status (*p* = 0.73), insurance type (*p* = 0.07), primary implant diagnosis (*p* = 0.25) or revision/explant (*p* = 0.42) between the female HF-SCS and LF-SCS patients.

Seventy males were implanted with LF-SCS and 54 with HF-SCS. There were more Hispanic patients in the HF-SCS group (14.8% vs 5.7%, *p* = 0.03). LF-SCS patients had more private insurance compared to HF-SCS (14.3% vs 3.7%), while HF-SCS had more Medicare (27.8% vs. 15.7%) or other governmental insurance (13% vs. 2.9%, *p* = 0.018). LF-SCS patients had more FBSS (45.7% vs 35.2%) and CRPS (14.3% vs 5.6%), while non-surgical refractory back pain was more common in HF-SCS (16.7% vs 1.4%, *p* = 0.006).

HF-SCS and LF-SCS males had improved VAS scores at 3 months (HF-SCS -2.48, 95%CI -3.46-(− 1.5), *p* < 0.001; LF-SCS -1.81, 95%CI -2.78-(− 0.84), *p* < 0.001) compared to baseline, which persisted to 6 months only for HF-SCS (HF-SCS -1.64, 95%CI -2.96-(− 0.33), *p* = 0.018; LF-SCS -0.98, 95%CI − 2.07-0.1, *p* = 0.074), and did not survive to 12 months (HF-SCS *p* = 0.22; LF-SCS *p* = 0.27), with no differences across groups (Fig. 3b).

PPR was improved among HF-SCS and LF-SCS males post-implantation (HF-SCS 11.84, 95%CI 0.03–23.65, *p* = 0.049; LF-SCS 15.54, 95%CI 2.34–28.73, *p* = 0.023) and at 3 months (HF-SCS 24.07, 95%CI 7.21–40.93, *p* = 0.009; LF-SCS 15.33, 95%CI 1.38–29.29, *p* = 0.033) compared to baseline (Fig. 3d). Furthermore, HF-SCS males had significantly better PPR at 3 (14.18, 95%CI 0.77–27.58, *p* = 0.034) and 6 months (19.6, 95%CI 2.88–36.32, *p* = 0.027) compared to LF-SCS males which did not survive to 12 months (HF-SCS *p* = 0.19; LF-SCS *p* = 0.18; *p* = 0.23 across groups).

LF-SCS males had significant reduction it opioid use at 6 months (LF-SCS -50.1, 95%CI -90.38-(− 9.83), *p* = 0.017) compared to baseline, but this was not present at other time points, or among HF-SCS patients at any timepoint. In addition, HF-SCS males required significantly less opioids compared to LF-SCS post-implantation (− 73.93, 95%CI -113.57-(− 34.29), *p* = 0.006), and at 6 months (− 81.34, 95%CI -114.95-(− 47.74), *p* = 0.01) but not at 3 (*p* = 0.19) and 12 months (*p* = 0.21, Fig. 3e). There were no differences in age (*p* = 0.19), marriage status (0.79), revision/explant (*p* = 0.56) between the male HF-SCS and LF-SCS patients.

### Linear mixed model

In the linear mixed model including age, sex, stimulator type, and time, VAS decreased with age (− 0.015, 95% CI -0.0003-(− 0.03), *p* = 0.047), at each time point, with the strongest effects seen at 3 months, (post implantation − 0.14, 95%CI -0.21-(− 1.16), 3 months − 1.1, 95%CI -1.09-(− 2.12); 6 months − 1.0, 95%CI -0.33-(− 1.41), 12 months − 0.26, 95%CI -0.24-(− 1.43), *p* < 0.001), and a trend towards increasing for female sex (0.44, 95%CI -0.003-0.88, *p* = 0.053), with a trend towards significant interaction between stimulator type and time (*p* = 0.068).

In the same model for PPR, 3 months (4.23, 95%CI 1.71–7.78) and 6 months (6.17, 95%CI 2.37–9.99) were associated with higher PPR (*p* < 0.001), while HF-SCS was associated with lower PPR (− 4.95, 95%CI -8.36- (− 1.5), *p* = 0.005), and there was a significant interaction between stimulator type and time (*p* = 0.027).

Finally, in the same model for opioid use, HF-SCS was associated with lower opioid use (− 70.02, 95%CI -23.75- (− 116.294), *p* = 0 < 0.001), without significant effect of age, sex, time or the interaction between time and stimulator type.

## Discussion

This single center retrospective study found significant improvement in VAS at 3 and 6 months which is no longer present at 12 months, possibly due to waning effects or loss to follow-up. LF-SCS and HF-SCS patients had improved PPR post-implantation and at 3 months, which persisted to 6 and 12 months for HF-SCS patients only. Furthermore, patients with HF-SCS had better pain control according to PPR at 3 and 6 months compared to the LF-SCS group. Some of these differences might be attributed to sex as only HF-SCS females had improved PPR at 3 and 6 months compared to baseline, while both HF-SCS and LF-SCS males had improvement post-implantation and at 3 months. Furthermore HF-SCS males had significantly better PPR at 3 and 6 months compared to LF-SCS males while this was only present at 6 months for females. Overall LF-SCS patients used significantly more opioids at each time point compared to those with HF-SCS; possibly due to the predominance of LF-SCS implantation prior to HF-SCS FDA approval in 2015 and recommendations for decreasing opioid prescribing in 2016. However, LF-SCS males used less opioids at 6 months, further indicating some differences in sex-based pathways. Finally, on linear mixed model analyses, including age, sex and stimulator type, VAS decreased with age and at each timepoint, with a trend towards increasing with female sex, which could be due to decreased pain perception in older age and due to the pain-relieving effects of SCS, while PPR increased at 3 and 6 months, which is likely due to the effects of the SCS, and opioid use was decreased with HF-SCS use, which could be related to HF-SCS efficacy and side effect profile compared to LF-SCS (Lautenbacher et al. 2017).

Similarly to another retrospective SCS study, we did not find sustained decrease in VAS at the 12 month endpoint (DiBenedetto et al. 2018). This is in contrast to prior clinical trials which note consistent decrease in VAS up to twelve (Kapural et al. 2015; De Andres et al. 2017) and twenty-four months (Kumar et al. 2008). This difference may be due to clinical trial methodology and patient population recruited for these trials. While we do not have patient satisfaction data, prior studies demonstrate high satisfaction even without long-term SCS efficacy (Kemler et al. 2008).

Next, when compared to LF-SCS, patients with HF-SCS had significant improvement in PPR, but not VAS at 3 and 6 months. These findings are in agreement with some smaller studies. De Andres et al. found no difference in pain numerical rating scale score at all time points, although the study was limited to FBSS patients (De Andres et al. 2017). A crossover study also found no difference between LF-SCS and paresthesia-free SCS although this study utilized 1 kHz paradigms rather than 10khz (Duse et al. 2019). In contrast, a randomized controlled trial found significantly greater decrease in VAS for HF-SCS compared to LF-SCS persisting to 12, which was extended to 24 months with the same finding (Kapural et al. 2015; Kapural et al. 2016). While other studies did not evaluate PPR, we used it in addition to VAS in order to distinguish from other potential pain the patient may be having at the time of visit, unrelated to the focus of SCS treatment (e.x. migraine). This difference in pain response to LF-SCS and HF-SCS may be somewhat attributed to sex, as supported by significant decreases in PPR among females implanted with HF-SCS but not LF-SCS. Furthermore, when accounting for age, time and stimulator type, there was a trend for higher VAS among females. Prior literature reported that females were more likely to have an SCS explanted than males, due to inadequate pain relief and possibly worsening depression associated with pain (Bretherton et al. 2021; Slyer et al. 2019).

However, few studies have reported on sex-based differences in SCS efficacy. Kumar et al., found that females had better chances of SCS trial success, and improved pain relief in the first year; however, long-term, males had a higher success rate (Kumar et al. 2006b). In contrast, a case series reported a trend towards a greater percentage of females finding relief from SCS but this difference was not statistically significant (Fiume et al. 1995). Similarly, a recent retrospective study found no differences in pain relief between males and females implanted with HF-SCS (Bretherton et al. 2021). Most recently, Mekhail et al. found no differences in self-reported pain by sex at 6 and 12 months following SCS implantation (Mekhail et al. 2021). Furthermore, a meta-analysis including 59 studies found no differences in pain relief according to sex (Taylor et al. 2014). The sex differences found here might be attributed to variances in the physiologic effects of HF-SCS and LF-SCS as yet discovered sex specific mechanisms, i.e., through hormones (Sherman and LeResche 2006) and immune mediators that suggest that males and females have different underlying pathophysiology of chronic pain (Sorge et al. 2015).

Lastly, while opioid use was higher among patients with LF-SCS, opioid dosage didn’t change significantly among HF-SCS or LF-SCS throughout the observed period. This is in contradiction with results from studies that found not only a decrease in opioid use in over half of patients, but apparently also found that approximately 30% of patients stopped taking opioids all together (Al-Kaisy et al. 2014; Van Buyten et al. 2013; DiBenedetto et al. 2018). Prior studies found that decrease in opioid use is traditionally greater among HF-SCS patients (Kapural et al. 2015) and is estimated to be around 25 morphine mili-equivalents (DiBenedetto et al. 2018); however this difference in opioid use decrease was not significant in a meta-analysis (Pollard et al. 2019). Furthermore, a recent study suggests that opioid use continues to significantly decrease beyond 12 months (Feng et al. 2021). It is unclear why our patient population did not experience a decrease in opioid dosage or why HF-SCS patients were on a significantly lower dose of opioids from the beginning; however it is possible that this is in part due to LF-SCS being an older patient cohort (prior to FDA approval of HF-SCS), in particular patients who were routinely treated with higher dosages prior to the opioid epidemic.

There are several limitations to this study. First, this study was conducted at a single tertiary-care institution, and there may be site specific baseline differences among this cohort. Next, we utilized self-reported VAS as a measure of efficacy and did not have the ability to look at functional status that may be a better representation of both objective and subjective components of pain (Gloth 3rd et al. 2001). Furthermore, not all patients filled out VAS scores and PPR at every visit; however we believe these data are missing at random. Third, we extracted morphine mili-equivalent doses from our electronic health record, and it is possible that the patients may have had additional outside opioid prescriptions, both prior to and following SCS implantation. Furthermore, opioid prescribing significantly changed following the 2016 Centers for Disease Control statement advising a maximum dose of 90 MME, resulting in increased rates of tapering, especially among women and those prescribed higher doses (Fenton et al. 2008). Given the FDA approval of HF-SCS in 2015, this likely did not significantly affect the findings among HF-SCS patients (Kapural et al. 2016). Finally, we did not assess for differences between implanters.

Some strengths of this study include a real-world analysis of SCS over 10 years that reflect clinical practice, stratified both by sex and type of stimulator. Despite this being a single center study, our population size is comparable to those seen in seminal SCS studies.

## Conclusion

Across all patients, HF-SCS was more effective at improving PPR and was associated with less opioid use compared to LF-SCS. Females with HF-SCS demonstrated significant improvement in PPR at 3 and 6 months compared to baseline, while this was not seen in LF-SCS females. In addition, among males PPR was significantly better for HF-SCS at 3 and 6 months while this was only present at 6 months for HF-SCS females. Finally, LF-SCS males used more opioids post-implantation and at 6 months, while LF-SCS females used more opioids post-implantation, at 3 and 6 months, potentially indicating some differences in sex-based pathways. Further studies are needed to evaluate the impact of SCS paradigm on pain control and opioid use between the two sexes.

## Supplementary Information

**Additional file 1.**

## Data Availability

The dataset analyzed in this study is available from the corresponding author on reasonable request.

## Abbreviations

SCS: Spinal Cord Stimulation
LF-SCS: Low Frequency Spinal Cord Stimulation
HF-SCS: High Frequency Spinal Cord Stimulation
VAS: Visual Analog Scale
PPR: Perceived Pain Reduction
FBSS: Failed Back Surgery Syndrome
CRPS: Complex Regional Pain Syndrome
