# Supplementary material for: Sex-specific differences in the efficacy of traditional low frequency versus high frequency spinal cord stimulation for chronic pain
Source: Bioelectron Med. 2022 Apr 28;8:8. doi: 10.1186/s42234-022-00090-2 (PMC9052649; doi:10.1186/s42234-022-00090-2)

**SUPPLEMENTAL TABLES**

Supplemental Table 1. Raw values of visual analog scale scores, perceived pain reduction and opioid use by type of stimulator. P-values are based on paired t-test between baseline and each individual time point based on stimulator type. *p-value compares HF-SCS to LF-SCS at a given time point

|  | HF-SCS | p-value | LF-SCS | p-value | *p-value across |
| --- | --- | --- | --- | --- | --- |
| VAS Scores (mean (SD)) |  |  |  |  |  |
| Baseline | 6.61 (1.85) |  | 6.31 (2.09) |  | 0.26 |
| Post-Implantation | 6.01 (2.22) | 0.09 | 5.73 (2.34) | 0.061 | 0.47 |
| 3 Months Post Implant | 4.25 (2.3) | **<0.001** | 4.87 (2.48) | **<0.001** | 0.18 |
| 6 Months Post Implant | 4.91 (2.42) | **<0.001** | 5.71 (2.17) | **0.005** | 0.127 |
| 12 Months Post Implant | 5.65 (2.39) | 0.16 | 5.69 (1.87) | 0.15 | 0.81 |
| Perceived Pain Reduction (mean (SD)) |  |  |  |  |  |
| Baseline | 31.74 (26.31) |  | 31.21 (29.91) |  | 0.99 |
| Post-Implantation | 55.41 (27.54) | **0.006** | 48.17 (29.84) | **<0.001** | 0.09 |
| 3 Months Post Implant | 61.18 (25.35) | **<0.001** | 47.93 (28.12) | **0.004** | **0.008** |
| 6 Months Post Implant | 58.38 (26.54) | **0.003** | 33.70 (25.94) | 0.09 | **<0.001** |
| 12 Months Post Implant | 57.17 (28.44) | **0.01** | 42.50 (26.75) | 0.052 | 0.067 |
| Morphine mili-Equivalents (mean (SD)) |  |  |  |  |  |
| Baseline | 39.47 (58.92) |  | 105.44 (118.49) |  | **<0.001** |
| Post-Implantation | 32.84 (49.39) | 0.99 | 107.91 (142.99) | 0.8 | **<0.001** |
| 3 Months Post Implant | 41.22 (91.67) | 0.26 | 110.23 (197.08) | 0.35 | **0.027** |
| 6 Months Post Implant | 14.66 (21.65) | 0.08 | 112.98 (142.97) | 0.79 | **<0.001** |
| 12 Months Post Implant | 32.66 (48.18) | 0.15 | 106.03 (146.08) | 0.36 | **0.014** |

Supplemental Table 2. Raw values of visual analog scale scores, perceived pain reduction and opioid use by type of stimulator. P-values are based on paired t-test between baseline and each individual time point based on stimulator type. *p-value compares HF-SCS to LF-SCS for the specific sex at a given time point

|  | Female | | | | | Male | | | | |
| --- | --- | --- | --- | --- | --- | --- | --- | --- | --- | --- |
|  | HF-SCS | p-val | LF-SCS | p-val | p-val* | HF-SCS | p-val | LF-SCS | p-val | p-val* |
| VAS Scores (mean (SD)) |  |  |  |  |  |  |  |  |  |  |
| Baseline | 6.77 (2.03) |  | 6.62 (2.13) |  | 0.67 | 6.49 (1.73) |  | 5.93 (2.01) |  | 0.13 |
| Post-Trial | 6.00 (2.46) | 0.13 | 5.78 (2.44) | **0.04** | 0.68 | 6.01 (2.05) | 0.139 | 5.68 (2.24) | 0.74 | 0.52 |
| 3 Months Post Implant | 4.38 (2.32) | **<0.001** | 5.16 (2.56) | **0.001** | 0.26 | 4.14 (2.32) | **<0.001** | 4.46 (2.34) | **<0.001** | 0.66 |
| 6 Months Post Implant | 5.35 (2.03) | **0.023** | 6.03 (1.92) | **0.032** | 0.32 | 4.38 (2.78) | **0.018** | 5.32 (2.42) | 0.074 | 0.25 |
| 12 Months Post Implant | 5.89 (2.29) | 0.37 | 5.82 (1.91) | 0.35 | 0.74 | 5.35 (2.6) | 0.22 | 5.50 (1.83) | 0.27 | 0.99 |
| Perceived Pain Reduction |  |  |  |  |  |  |  |  |  |  |
| Baseline | 29.82 (30.23) |  | 28.65(30.54) |  | 0.96 | 34 (23.32) |  | 34.09 (29.38) |  | 0.96 |
| Post-Trial | 57.03 (32.26) | 0.053 | 45.11 (31.54) | **0.014** | 0.12 | 54.19 (23.50) | **0.049** | 51.10 (28.13) | **0.023** | 0.45 |
| 3 Months Post Implant | 60.71 (24.91) | **0.016** | 48.45 (26.63) | 0.053 | 0.12 | 61.47 (25.98) | **0.009** | 47.29 (30.26) | **0.033** | **0.034** |
| 6 Months Post Implant | 63.19 (28.02) | **0.022** | 3.54 (28.11) | 0.11 | **0.004** | 53.46 (24.61) | 0.09 | 33.86 (24.00) | 0.60 | **0.027** |
| 12 Months Post Implant | 59.64 (28.79) | 0.053 | 44.72 (29.28) | 0.18 | 0.22 | 53.33 (29.16) | 0.19 | 40.00 (24.29) | 0.18 | 0.23 |
| Morphine mili-Equivalents |  |  |  |  |  |  |  |  |  |  |
| Baseline | 28.52 (29.13) |  | 95.99 (93.76) |  | **0.003** | 49.7 (72.39) |  | 104.79 (129.93) |  | 0.053 |
| Post-Trial | 25.77 (25.3) | 0.78 | 103.84 (147.40) | 0.45 | **0.001** | 42.59 (65.11) | 0.25 | 112.16 (139.25) | 0.86 | **0.006** |
| 3 Months Post Implant | 28.93 (34.52) | 0.11 | 89.06 (99.04) | 0.48 | **0.006** | 58.54 (119.22) | 0.37 | 134.37 (267.58) | 0.87 | 0.19 |
| 6 Months Post Implant | 22.05 (30.7) | 0.45 | 129.1 (166.53) | 0.12 | **0.011** | 17.50 (19.45) | 0.21 | 96.52 (113.5) | **0.017** | **0.01** |
| 12 Months Post Implant | 25.69 (28.55) | 0.83 | 118.63 (140.99) | 0.071 | 0.064 | 39.62 (59.27) | 0.23 | 92.82 (151.8) | 0.10 | 0.21 |

**SUPPLEMENTAL FIGURES**

Supplemental Figure 1: Visual Analog Scale Pain Scores, Perceived Pain Reduction and Morphine Equivalent Daily Dose based on Type of Spinal Cord Stimulator. Blue stars note significant difference for HF-SCS at that time point compared to baseline. Yellow stars note significant differences for LF-SCS at that time point compared to baseline. Black stars note significant differences between HF-SCS and LF-SCS at that time point.

1a. Visual analog scale pain score distribution by type of stimulator post-implantation, 3 months after implant, 6 months after implant and 12 months after implant

1b. Perceived pain reduction distribution by type of stimulator post-implantation, 3 months after implant, 6 months after implant and 12 months after implant

1c. Opioid use in morphine miliequivalents by type of stimulator post-implantation, 3 months after implant, 6 months after implant and 12 months after implant


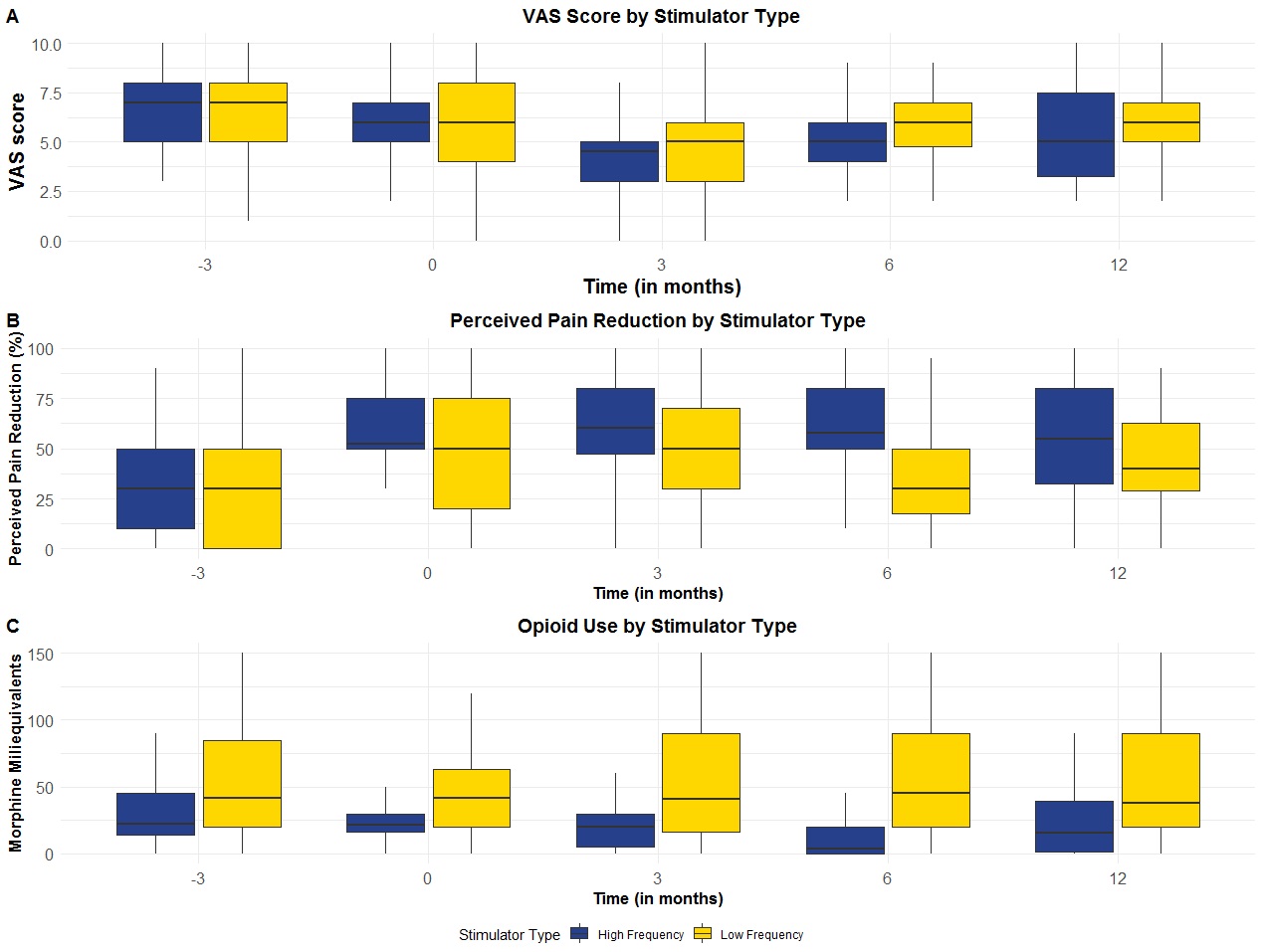


*

*

*

*

*

*

*

*

*

*

*

*

*

*

*

*

*

Supplemental Figure 2: Visual Analog Scale Pain Scores, Perceived Pain Reduction and Morphine Equivalent Daily Dose based on Type of Spinal Cord Stimulator and Sex. Blue stars note significant difference for HF-SCS at that time point compared to baseline. Yellow stars note significant differences for LF-SCS at that time point compared to baseline. Black stars note significant differences between HF-SCS and LF-SCS at that time point.

2a. Visual analog scale pain score distribution by type of stimulator post-implantation, 3 months after implant, 6 months after implant and 12 months after implant in females

2b. Visual analog scale pain score distribution by type of stimulator post-implantation, 3 months after implant, 6 months after implant and 12 months after implant in males

2c. Perceived pain reduction distribution by type of stimulator post-implantation, 3 months after implant, 6 months after implant and 12 months after implant in females

2d. Perceived pain reduction distribution by type of stimulator post-implantation, 3 months after implant, 6 months after implant and 12 months after implant in males

2e. Opioid use in morphine miliequivalents by type of stimulator post-implantation, 3 months after implant, 6 months after implant and 12 months after implant in females

2f. Opioid use in morphine miliequivalents by type of stimulator post-implantation, 3 months after implant, 6 months after implant and 12 months after implant in males


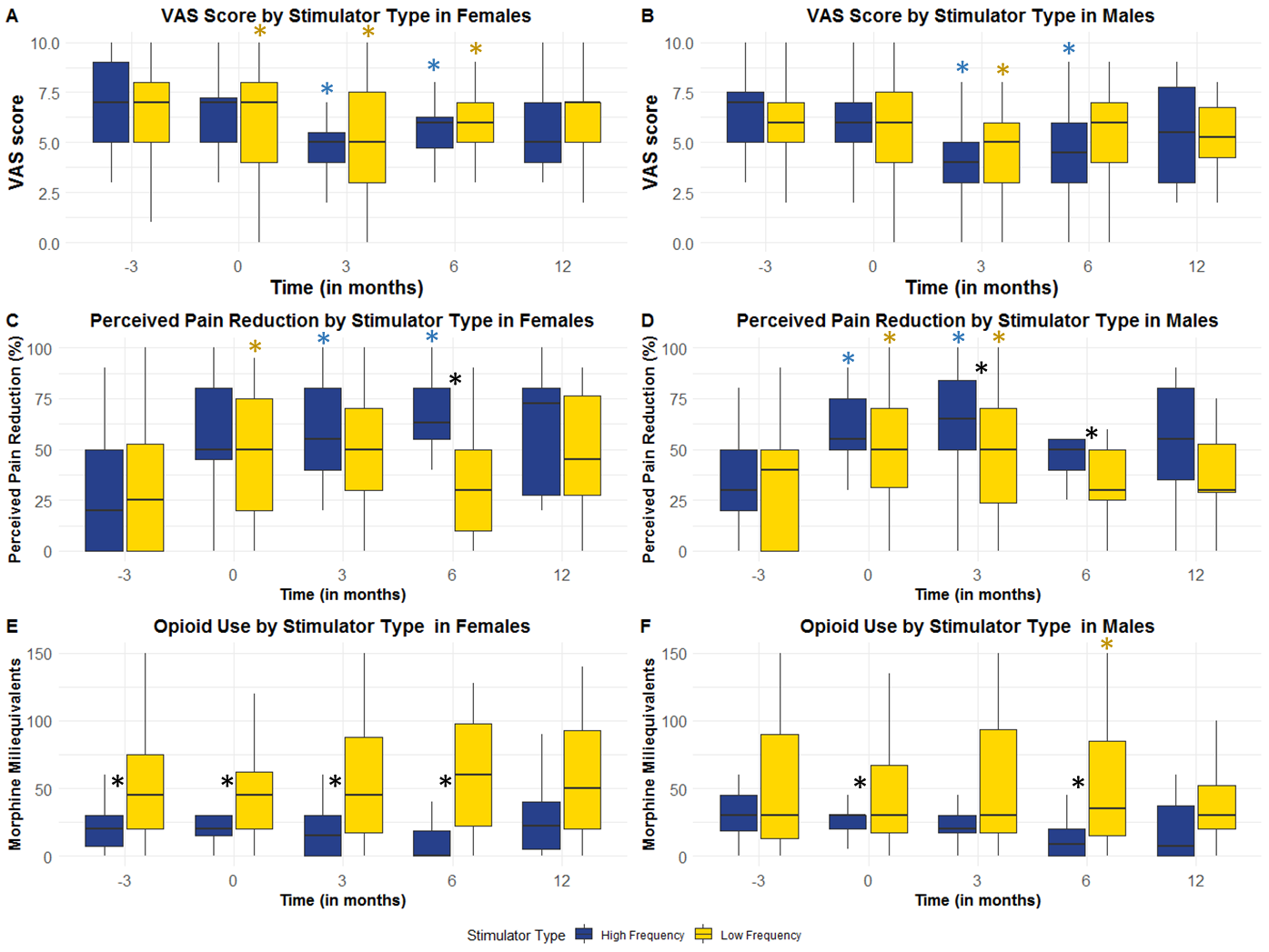

Supplement: Supplementary file 1 — Additional file 1. [file 42234_2022_90_MOESM1_ESM.docx]
